# Fabrication of ZnO Nanowire Cold Cathode Flat-Panel X-ray Source with a Reflective Anode

**DOI:** 10.3390/nano14181504

**Published:** 2024-09-16

**Authors:** Chengyun Wang, Guofu Zhang, Qi Liu, Song Kang, Shaozhi Deng, Jun Chen

**Affiliations:** State Key Laboratory of Optoelectronic Materials and Technologies, Guangdong Province Key Laboratory of Display Material and Technology, School of Electronics and Information Technology, Sun Yat-Sen University, Guangzhou 510275, China; wangchy75@mail2.sysu.edu.cn (C.W.); zhanggfu@mail.sysu.edu.cn (G.Z.); liuq558@mail2.sysu.edu.cn (Q.L.); kangs5@mail2.sysu.edu.cn (S.K.); stsdsz@mail.sysu.edu.cn (S.D.)

**Keywords:** ZnO nanowire, cold cathode, reflective anode, flat-panel X-ray source

## Abstract

A novel reflective anode flat-panel X-ray source using ZnO nanowire cold cathode and a metal anode has been developed. Simulation analysis indicated that the reflective anode structure reduces electric field concentration compared to a transmission anode structure. The current–voltage characteristics, X-ray radiation dose rate, and stability of the fabricated device were thoroughly characterized. The device demonstrated a maximum emission current of 481.1 μA and a maximum radiation dose rate of 303 μGy/s at an anode voltage of 40 kV. The X-ray imaging of various objects was also conducted. Our findings are of significance for developing high-performance, robust flat-panel X-ray sources for diverse applications.

## 1. Introduction

X-ray imaging techniques are widely used in medical diagnostics, security screening, and industrial non-destructive testing, etc. [1,2,3,4,5,6]. However, conventional thermionic X-ray sources have inherent drawbacks, including high power consumption, large dimensions, and long startup times. In contrast, cold cathode X-ray sources can achieve low power consumption, compact size, and rapid response. Cold cathode X-ray sources using quasi-one-dimensional nanomaterials such as carbon nanotubes (CNTs) [7,8,9,10,11], silicon nanotips [12,13,14], and metal oxide nanowires [15,16,17] have been reported. Some products of carbon nanotube cold cathode X-ray sources have already been commercialized [18]. Despite these advancements, like conventional thermal cathode X-ray sources, cold cathode X-ray sources usually have one focus, which requires large source-to-object distance (SOD) for imaging. To address this issue, researchers have proposed a flat-panel X-ray sources (FPXSs) by integrating multiple micro-X-ray sources into a planar array [19,20,21,22]. In recent years, cold cathode FPXSs based on ZnO nanowires have been successfully fabricated, including both diode and triode structures [17,23,24,25,26,27]. The diode structure allows for large-area irradiation, making it suitable for applications such as digital radiography (DR) imaging, sterilization, and electrostatic discharge control [28,29,30]. The triode structure, with its additional gate electrode enabling localized emission, shows significant application potential in conformal and low-dose stationary computed tomography (CT) imaging when combined with novel algorithms [31,32,33,34,35].

Currently reported cold cathode FPXSs, whether in diode or triode structure, use a thin transmission anode made of metal thin film. During operation, electrons from the cathode bombard the front of the transmission anode target, and X-rays are emitted from the back of the anode. To achieve higher emission efficiency, the thickness of the anode’s metal thin film is typically at the micrometer scale. However, these thin conductive films serve as both the anode target and high-voltage connection leads, and they are susceptible to damage caused by high-voltage-induced arcing or prolonged electron bombardment, which severely shorten the lifetime of the device. In contrast, the reflective anode structure used in conventional thermionic X-ray sources, which has been developed and optimized for many years, is more robust and exhibits exceptional stability and reliability [36,37,38].

In this study, a novel reflective anode FPXS using ZnO nanowire cold cathode is fabricated. Its electrical characteristics and X-ray emission performance were studied. This preliminary work demonstrated the feasibility of this reflective anode FPXS.

## 2. Device Structure and Fabrication

**Figure 1:**
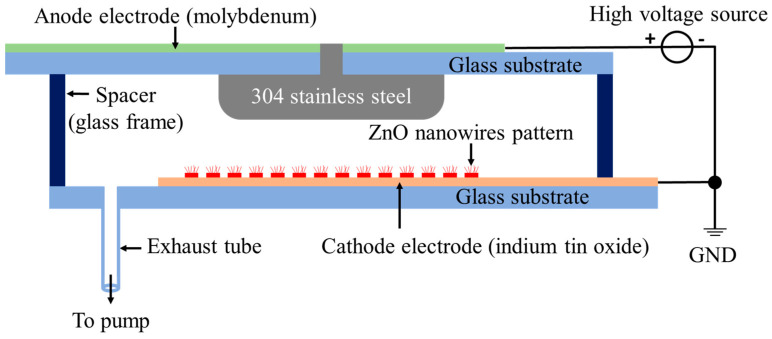
Schematic diagram of the structure of the reflective anode FPXS.

The structure of the reflective anode FPXS is illustrated in Figure 1, which consists primarily of a cathode panel and an anode panel. In this study, the cathode panel is made from a 95 mm × 124 mm × 3 mm glass substrate, onto which a 500 nm−thick indium tin oxide (ITO) cathode electrode and ZnO nanowire field emitter arrays (FEAs) are fabricated. The FEAs consist of circular patterns with a 5 μm diameter, arranged in a square array with a 30 μm spacing, centrally positioned on the ITO electrode. The anode panel consists of a 304 stainless steel anode measuring 30 mm × 30 mm × 6 mm, adhered to a separate 78 mm × 124 mm × 3 mm glass substrate. On the reverse side of this substrate, a 500 nm−thick molybdenum (Mo) anode electrode is deposited. To ensure effective insulation and sealing, a 10 mm−thick glass frame serves as an insulating spacer between the cathode and anode panels. Additionally, an exhaust tube is installed on the cathode glass substrate to connect to an external vacuum system, maintaining the required internal vacuum environment.

The ZnO nanowire FEAs are fabricated using microfabrication techniques [39]. The main steps are outlined as follows. Initially, the glass substrate is sequentially cleaned with acetone, anhydrous ethanol, and deionized water via ultrasonic treatment to remove surface contaminants. Subsequently, the ITO cathode electrode is deposited on the cleaned substrate using a shadow mask technique combined with magnetron sputtering equipment. The ITO-coated glass substrate is then annealed in a tube furnace with a maximum temperature of 470 °C. Following this, a regular array of Zn patterns is fabricated using photolithography and electron beam evaporation techniques, followed by growth of ZnO nanowire arrays using thermal oxidation in a tube furnace [40]. The anode electrode is prepared using magnetron sputtering. After preparing both anode and cathode substrates, they are sealed using low-melting-point glass frit, leaving only the exhaust tube on the cathode substrate open to be connected to the pumping system.

The morphology of the ZnO nanowire FEAs on the cathode panel were characterized using a scanning electron microscope (SUPRA 55, Carl Zeiss AG, Oberkochen, Germany). Following the sealing of both the cathode and anode panels, a vacuum was established inside the device by pumping through the exhaust tube using a combination of mechanical and molecular pumps. The pressure was 2 × 10^−5^ Pa during testing. The field emission characteristics of the device were measured using a high-voltage DC power supply (TD2202, Teslaman Technology, Dalian, China) and a multimeter (UT71A, UNI-Trend Technology, Seoul, Republic of Korea). Radiation dose was measured with an X-ray dosimeter (MagicMax, IBA Dosimetry, Bartlett, IL, USA), X-ray spectra were recorded using an X-ray spectrometer (X-123 CdTe, AMETEK, Oak Ridge, TN, USA), and X-ray transmission imaging results were captured using a flat-panel imaging detector (AXIOS-1515, Teledyne DALSA, Waterloo, ON, Canada).

## 3. Simulation Analysis

**Figure 2:**
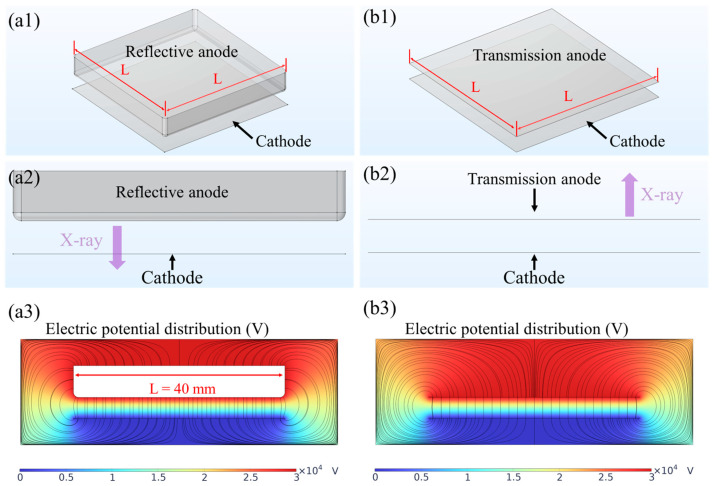
COMSOL simulations of reflective and transmission anode FPXS. (**a1**–**a3**) Reflective structure: 3D view, cross section, and electric potential distribution; (**b1**–**b3**) transmission structure: 3D view, cross section, and electric potential distribution.

The electric field distribution in a diode structure plays a crucial role in device performance and reliability. Since high-voltage-induced arcing can easily damage the device, a structure with a lower localized electric field is preferred. To compare the electric field distributions in reflective and transmission anode FPXS, finite element simulations were performed using COMSOL Multiphysics. Figure 2 illustrates the geometric configurations and cross-sectional electric potential distributions of the simulated devices. Simulation parameters were set as follows: (1) anode voltage: 30 kV; (2) anode-cathode gap: 4 mm; (3) anode thickness: 6 mm for reflective type and 1 μm for transmission type; (4) cathode electrode thickness: 0.5 μm; and (5) cathode side length: 40 mm. The side length of the anode electrode, denoted as L, was systematically varied to analyze the electric field distributions for different anode dimensions. This approach aimed to identify optimal structures and parameters that favor stable FPXS operation under high-anode-voltage conditions. Particular attention was given to regions near electrode edges and corners, where field enhancement effects are most pronounced and maximum electric field intensities are typically observed.

**Figure 3:**
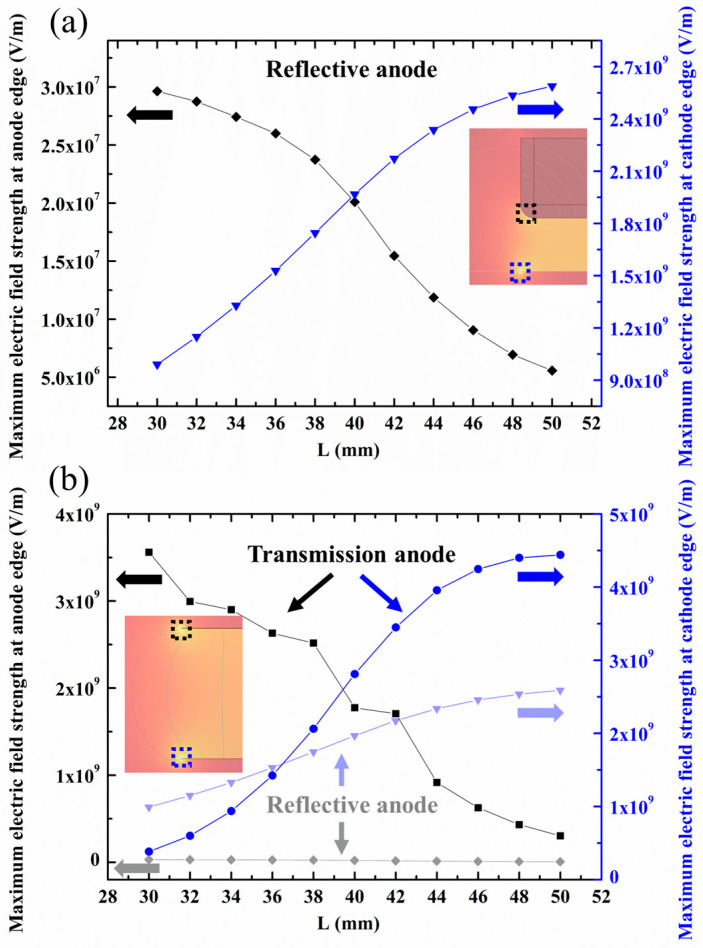
Simulated results of maximum electric field strength at the edges of the anode and cathode versus anode side length L for (**a**) reflective anode, and (**b**) transmission anode. The results for the reflective anode are superimposed to facilitate direct comparison.

Figure 3 illustrates the relationship between the maximum electric field strength at the electrode edges and the anode electrode side length L for both structures. The simulation results reveal comparable maximum electric field strengths near the cathode edges for both reflective and transmission anode FPXS. The maximum field strength at the cathode edge for both the reflective and transmission anode FPXS are all in the range of 3.8 × 10^8^ V/m–4.4 × 10^9^ V/m. For L less than 38 mm, the reflective anode FPXS exhibits slightly higher maximum field strength (9.9 × 10^8^ V/m–1.5 × 10^9^ V/m) at the cathode edge compared to the transmission anode FPXS (3.8 × 10^8^ V/m–1.4 × 10^9^ V/m), which is attributed to the additional field contribution from the side surface of the thick reflective anode. As L increases, this trend reverses, with the transmission FPXS eventually showing marginally higher field strengths at the cathode edge. At L = 50 mm, the maximum electric field strength at the cathode edge for the transmission anode FPXS is approximately 4.4 × 10^9^ V/m, while that of the reflective anode FPXS is about 2.6 × 10^9^ V/m.

However, a significant difference is observed at the anode edges. At the anode edge, the reflective anode FPXS consistently demonstrates electric field strengths two orders of magnitude lower (in the 10^7^ V/m range) compared to the transmission FPXS (in the 10^9^ V/m range). This substantial difference is primarily due to the thin edge of the transmission target, which dramatically enhances the field concentration. Conversely, the thicker anode with rounded edges in the reflective structure mitigates this effect. With further increase in L, maximum field strengths at anode edges decrease for both structures, ultimately changing slowly with L due to the large distance, and converging to a finite value.

This finding suggests that adopting a reflective anode structure may potentially reduce the risk of vacuum breakdown induced by the high anode voltage. Consequently, FPXSs with reflective anode structures could achieve better stability and higher reliability when the device operates under high anode voltage.

## 4. Results of Device Characterization

### 4.1. Morphological Characterization

**Figure 4:**
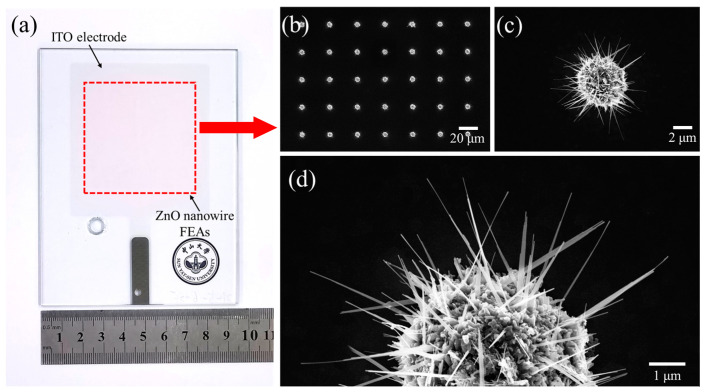
Optical image of the cathode panel and SEM images of ZnO nanowire FEAs. (**a**) Optical image of the cathode panel; (**b**) SEM of ZnO nanowire FEAs; (**c**) SEM of a single ZnO nanowire pattern; (**d**) magnified SEM of (**c**).

The optical image of the device cathode panel is shown in Figure 4a, where the light-gray transparent film represents the ITO electrode, with the central area outlined by the red dashed box indicating the ZnO nanowire FEAs. Due to its wide bandgap (approximately 3.37 eV), ZnO demonstrates good transparency in the visible light range, allowing this area to appear transparent to the naked eye. SEM characterization results of the nanowire array at the central point in this area are shown in Figure 4b–d. Statistical analysis of these SEM images revealed that the average length of the nanowires was approximately 4 μm, with tip diameters around 22 nm, and a growth density of approximately 3.8 μm^−2^.

**Figure 5:**
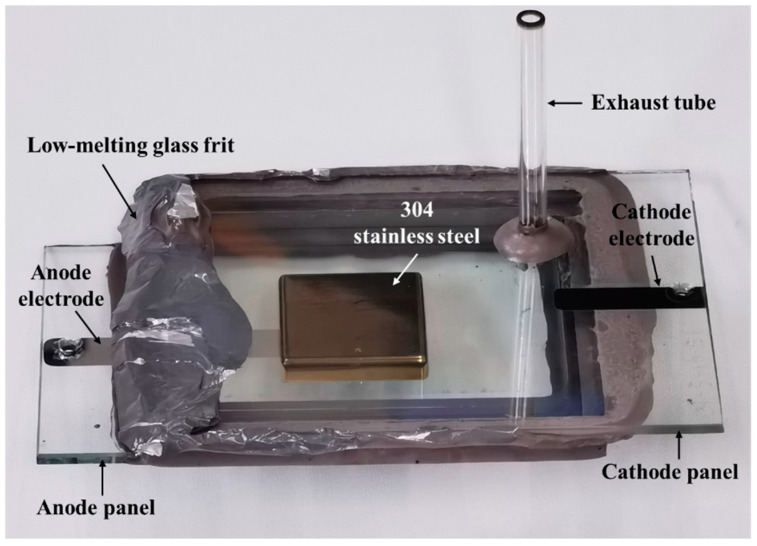
Optical image of the reflective anode FPXS.

Figure 5 shows the optical image of the device obtained after sealing the cathode and anode panels. Since the cathode substrate is transparent, the 304 stainless steel anode can be directly observed when viewing from the cathode substrate towards the anode direction.

### 4.2. I−V Characteristics

**Figure 6:**
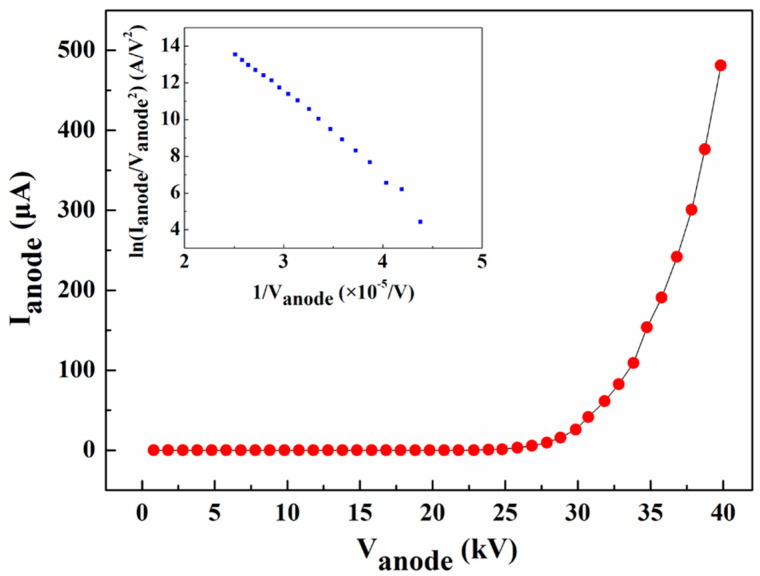
I−V characteristic curve and F−N plot of the reflective anode FPXS.

The obtained current–voltage (I−V) curve and the corresponding Fowler−Nordheim (F−N) plot are shown in Figure 6. It can be observed that the maximum field emission current of approximately 481.1 μA is achieved at an anode voltage of 40 kV, corresponding to a current density of about 0.59 mA/cm^2^ (calculated based on the ZnO nanowire pattern area of 0.81 cm^2^ directly beneath the anode). The F−N plot shown in the inset approximates a straight line with a negative slope, suggesting that the current is indeed from field-induced electron emission.

### 4.3. X-ray Emission Characteristics

**Figure 7:**
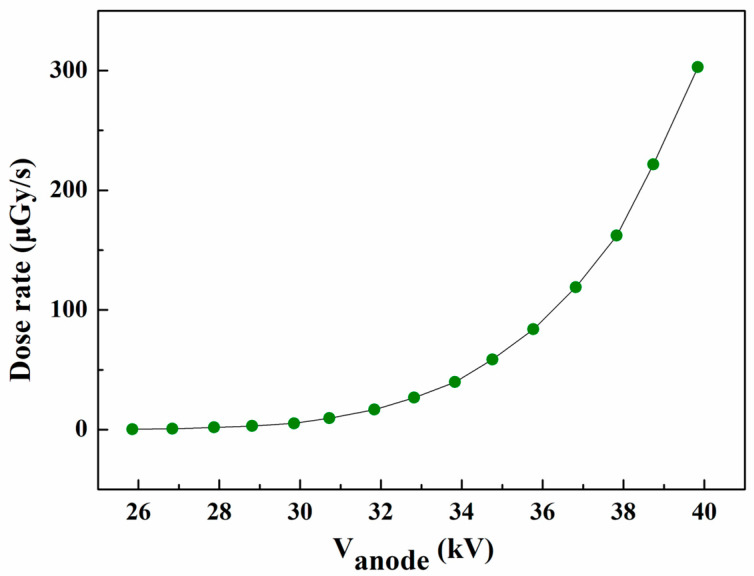
Dose rate–anode voltage curve of the reflective anode FPXS.

We collected the dose rate data of the X-rays emitted by the reflective anode FPXS at different anode voltages, which were measured 10 cm from the surface of the anode. The curve of the dose rate data is shown in Figure 7. It can be observed that the X-ray dose rate increases with increasing anode voltage, with the maximum dose rate of approximately 303 μGy/s is measured at an anode voltage of 40 kV.

**Figure 8:**
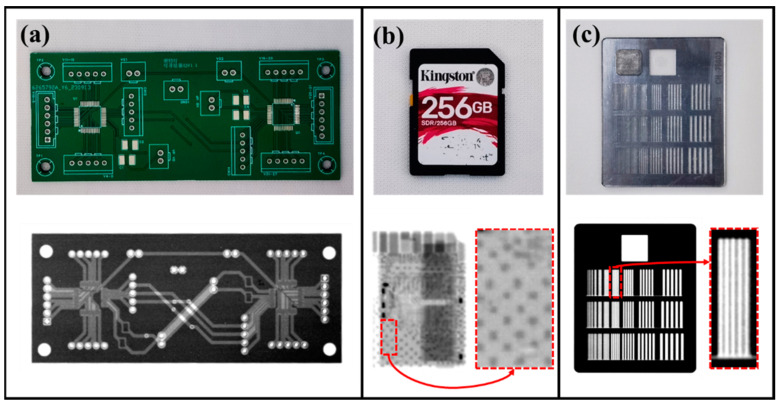
Optical (top) and corresponding X-ray (bottom) images obtained using the reflective anode FPXS. (**a**) PCB; (**b**) memory card; (**c**) line-pair resolution test card.

X-ray imaging experiments were carried out using the fabricated reflective anode FPXS. A flat-panel X-ray detector was placed 40 cm from the surface of the device’s metal anode, and the objects to be imaged (a printed circuit board (PCB), a memory card and a line-pair card), were positioned on the detector surface. The anode voltage of the device was set to 37 kV, and the objects were exposed for 10 s. Figure 8a–c show the X-ray imaging results. The imaging results clearly reveal the external contours and internal traces of the PCB, as well as the external pins and internal flash memory chip of the memory card. Additionally, the X-ray images of the line-pair card showed that the finest resolvable line pair was 1.8 lp/mm.

### 4.4. Stability Characterization

**Figure 9:**
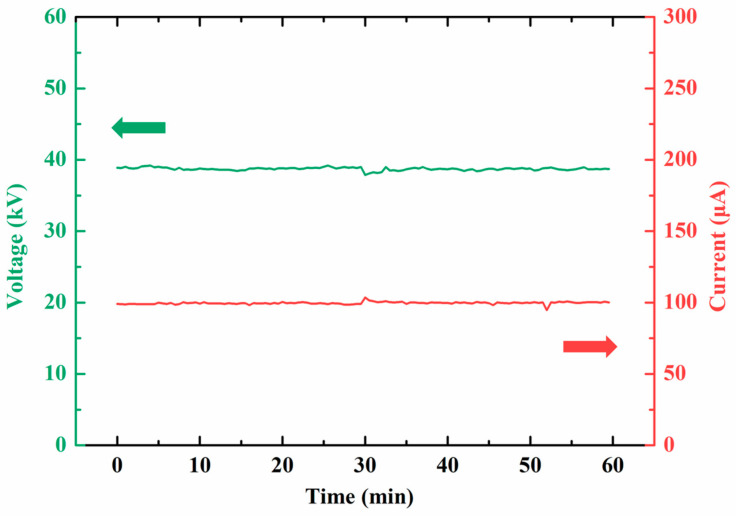
Voltage and current curves of reflective anode FPXS under 100 μA constant current operation mode.

We investigated the device’s stability using a reflective anode FPXS device operating at anode voltage of 39 kV and current of ~100 μA. The device was continuously driven by a high-voltage power supply under constant current mode. We collected 120 data points over a 60 min period, sampling every 30 s, and the results are illustrated in Figure 9. The recorded current and voltage curves indicate that under a constant current of 100 μA, the anode voltage remained stable at approximately 39 kV. The current and voltage fluctuations were calculated using Equations (1) and (2), respectively:

(1) $$\delta_{I}=\frac{\sum_{i=1}^{N}\left|I_{i}-\bar{I}\right|}{N\bar{I}}\times 100\%,$$

(2) $$\delta_{V}=\frac{\sum_{i=1}^{N}\left|V_{i}-\bar{V}\right|}{N\bar{V}}\times 100\%,$$

where N represents the number of data points in the curve, $I_{i}$ and $V_{i}$ are the current and voltage at each recorded point, and $\bar{I}$ and $\bar{V}$ are the average current and voltage, respectively. The current fluctuation $\delta_{I}$ was calculated to be 0.54% and the voltage fluctuation $\delta_{V}$ was 0.38%. These small fluctuations in both voltage and current confirm the excellent stability of the device. Furthermore, we measured the device temperature using an infrared thermometer and observed that the temperature reached 101.3 °C during operation, without any active cooling measures. This demonstrates the device’s inherent ability to maintain stable operation. With the addition of proper cooling solutions, the device’s stability performance could be further enhanced. In actual X-ray imaging applications, the X-ray source is typically operated for a short period, which is significantly shorter than the continuous operation time in our stability testing. The above results indicate that the device has potential for operating stably for at least several thousand exposure shots.

## 5. Conclusions

Simulation analysis reveals that the maximum electric field intensity at the edge of the reflective flat-panel X-ray source anode is significantly lower than that of a transmission-type structure, which helps reduce the risk of vacuum breakdown. Based on this, we developed a reflective-anode flat-panel X-ray source using ZnO nanowire cold cathode FEAs. At an anode voltage of 40 kV, the device achieved a maximum emission current of 481.1 μA and a maximum radiation dose rate of 303 μGy/s. X-ray imaging of objects and high stable operation were demonstrated. Our study verifies the feasibility of using reflective anode to achieve a highly stable cold cathode flat-panel X-ray source.

## Data Availability

Data are contained within the article.
